# Clinical Effectiveness of Biological Immunomodulators in SARS-CoV-2-Associated Multisystem Inflammatory Syndrome in Children: A Systematic Review

**DOI:** 10.3390/children11101180

**Published:** 2024-09-27

**Authors:** Ji Young Lee, Jimin Kim, Soo-Han Choi, Dong Hyun Kim, Ki Wook Yun, Yae-Jean Kim, Giang Pham Ha Cao, Miyoung Choi, Jong Gyun Ahn

**Affiliations:** 1 Department of Pediatrics, Severance Children’s Hospital, Yonsei University College of Medicine, Seoul 03722, Republic of Korea; sophielee0319@yuhs.ac; 2 Division for Healthcare Technology Assessment Research, National Evidence-Based Healthcare Collaborating Agency, Seoul 04933, Republic of Korea; jimin@neca.re.kr; 3 Department of Pediatrics, Pusan National University School of Medicine, Busan 49241, Republic of Korea; soohan_choi@pusan.ac.kr; 4 Department of Pediatrics, Inha University College of Medicine, Inha University Hospital, Incheon 22332, Republic of Korea; id@inha.ac.kr; 5 Department of Pediatrics, Seoul National University College of Medicine, Seoul 03080, Republic of Korea; pedwilly@snu.ac.kr; 6 Department of Pediatrics, Sungkyunkwan University School of Medicine, Samsung Medical Center, Seoul 06351, Republic of Korea; yaejeankim@skku.edu; 7 Department of Pediatrics, University of Medicine and Pharmacy, Ho Chi Minh 17000, Vietnam; drhagiang@ump.edu.vn; 8 Institute for Immunology and Immunological Disease, Yonsei University College of Medicine, Seoul 03722, Republic of Korea

**Keywords:** biological immunomodulators, pediatric multisystem inflammatory disease, SARS-CoV-2

## Abstract

Background: Although there is consensus to use immunoglobulins and corticosteroids as first-line treatments for multisystem inflammatory syndrome in children (MIS-C), the effectiveness of biological immunomodulators in patients refractory to standard therapy remains unclear. We aimed to outline real-world data on biological immunomodulators. Method: A literature search using Ovid-Medline, EMBASE, Cochrane CDSR, and KMBASE was conducted from September 2021 to August 2022; certainty of evidence was assessed via GRADE. Results: Among 258 studies, 10 were selected for analysis, of which 2 were observational studies (with control groups receiving standard therapy of either intravenous immunoglobulins and/or glucocorticoids) and 8 were single-arm studies. In all, 145 patients were treated with biological immunomodulators (anakinra (72; 49%) or infliximab (65; 44%)). In the first observational study, patients in the anakinra group initially exhibited a lower left ventricular ejection fraction than those in the control group. In the second study, patients in the infliximab group required less additional therapy and showed lower newly developed left ventricular dysfunction rate and reduced C-reactive protein levels. The clinical outcomes associated with each biological agent in single-arm studies were not reported individually. Conclusions: Biological immunomodulators are feasible therapeutic options for refractory MIS-C. Nevertheless, further research is warranted to demonstrate clinical efficacy.

## 1. Introduction

Hyperinflammatory shock after SARS-CoV-2 infection or exposure, also known as multisystem inflammatory syndrome in children (MIS-C), was first reported in April 2020 after the onset of the coronavirus (COVID-19) pandemic [1]. The pathogenesis of MIS-C, which is characterized by fever and multi-organ involvement, combined with a recent history of SARS-CoV-2 infection or exposure, still remains unclear. Studies have hypothesized that an interplay between post-inflammatory cytokine storm, adaptive immune responses involving both T and B cells, and autoimmune features contributes to the onset of MIS-C [2].

Based on the proposed mechanisms and general consensus, the treatment of MIS-C is similar to that of Kawasaki disease and involves the use of high-dose intravenous immunoglobulin (IVIg) and/or corticosteroid as first-line therapies [3]. When the condition is severe and the patient is refractory to first-line treatment, exhibiting persistent fever and/or end-organ involvement, a second dose of IVIg may be administered [4,5,6,7]. In this context, biological immunomodulators can be considered as an alternative therapeutic option. However, owing to the inadequate number of patients treated with biological immunomodulators, clinical evidence supporting their use as a second-line treatment is insufficient.

The main types of biological immunomodulators used to treat MIS-C include interleukin (IL)-1 inhibitors, IL-6 inhibitors, and anti-tumor necrosis factor (TNF) inhibitors. Elevated IL-1 levels have been observed in adult patients with severe COVID-19 [4]. Additionally, SARS-CoV-2-induced epithelial injury results in the release of IL-1β, which further triggers innate immune responses. IL-1 inhibitors can block this auto-inflammatory chain through inhibition of receptor binding (e.g., anakinra) or signaling pathway (e.g., canakinumab). Anakinra is licensed for the treatment of rheumatoid arthritis, cryopyrin-associated periodic syndrome, and cytokine release storms caused by chimeric antigen receptor T-cell therapy [3,4]. Currently, anakinra is the only IL-1 inhibitor used for the treatment of MIS-C. Administration of anakinra to patients with refractory MIS-C alleviates cytokine storm, demonstrating positive clinical outcomes [5]. Additionally, IL-6 inhibitors, such as tocilizumab (a monoclonal antibody against the IL-6 receptor), have been used to control cytokine storms and elevate Th1- and Th17-mediated innate immune responses [2,6]. Lastly, anti-TNF inhibitors, such as infliximab, have been administered to patients with MIS-C, as they are widely used for the treatment of rheumatoid arthritis, ankylosing spondylitis, inflammatory bowel disease (IBD), and refractory Kawasaki disease. However, evidence supporting the clinical efficacy of biological immunomodulators is insufficient.

Therefore, we conducted a systematic review on the clinical efficacy of biological immunomodulators to provide better clinical recommendations for their use in the treatment of MIS-C.

## 2. Materials and Methods

This study adhered to the guidelines of Preferred Reporting Items for Systematic Reviews and Meta-Analyses (PRISMA) (Protocol Registration No. CRD42023417146) (See Table S1 of Supplemental Materials).

### 2.1. Data Sources and Search Strategy

Ovid-Medline, EMBASE, Cochrane CDSR, and the Korean database KMBASE through September 2021 were systematically searched. Terms such as COVID-19, SARS-CoV-2, and multisystem inflammatory diseases were used. Ongoing trials and preprint articles were excluded. A manual search of the reference lists of relevant primary and review articles was also performed for completeness. As new observations on the main outcomes of COVID-19 are continuously being reported, the search was updated on the 10th day of each month, starting from November 2021 to August 2022. Ovid-MEDLINE was systematically searched for search updates, and the complete electronic search strategy for each database is presented in Supplementary Material.

### 2.2. Eligibility Criteria and Study Selection

Articles that matched the following requirements were considered: (1) patients were children or adolescents with MIS-C satisfying the age criteria established by the World Health Organization, the US Center for Disease Control, and/or other guidelines less than 20 years old; (2) interventions were undertaken using immunomodulators (IL-1, IL-6 inhibitors, and anti-TNF inhibitors); (3) the comparator was placebo or standard-of-care treatment; (4) outcomes included mortality, mechanical ventilation (MV), hemodynamic support, coronary artery aneurysm at discharge, abnormal cardiac function, and time to defervescence; (5) the study was designed as a randomized controlled trial (RCT), comparative study, and single-arm study. Studies conducted on patients over 20 years old and that used biological immunomodulators to treat acute COVID-19 infection were excluded. Only English and Korean studies were included in this analysis. Two review authors (JK and MC) independently and in duplicate evaluated the publications for inclusion based on title and abstract and then reviewed relevant full-text articles using Covidence. Disagreements during the review process were addressed by consensus, with the involvement of a third review author (JGA).

### 2.3. Data Extraction and Methodological Quality Assessment

**Figure 1:**
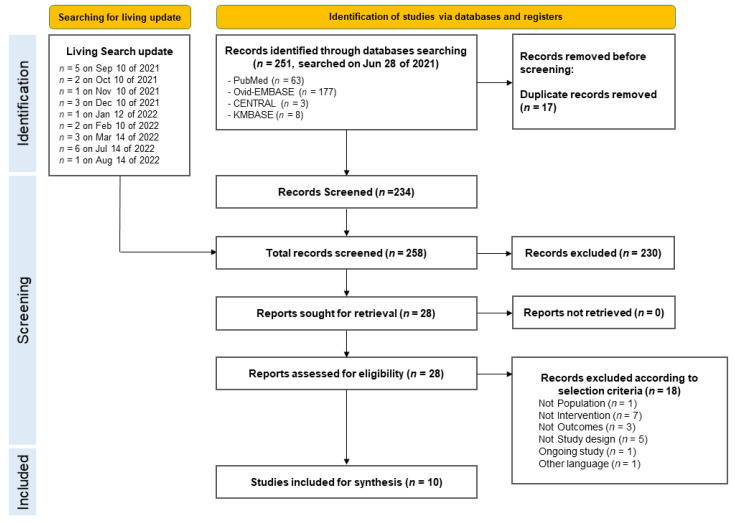
PRISMA flow chart of study selection.

Two authors (JK and MC) independently assessed the quality of the selected studies using the Cochrane risk of bias tool. Disagreements were resolved by consensus, with the participation of a third review author (JGA). Two authors (JK and MC) extracted information from each included trial. These evaluations were performed independently and yielded separate results. Disagreements were resolved through discussion and third opinion (JGA). The following information was included in the data extraction form: first author, publication year, study design, study subject characteristics, immunomodulatory type, and outcome (Figure 1).

The quality of the included observational studies was assessed using the RoBANS quality assessment tool, and the risk of bias is summarized in Figure S1 and Table S3 of Supplementary Materials. The case-series design did not assess the risk of bias. A case series was described as a series of at least five individuals who usually received the same intervention and included a non-control group.

### 2.4. Data Synthesis and Analysis

Due to the lack of consistency in the conceptualization and measurement of outcomes, a narrative synthesis is presented. Due to this heterogeneity, a meta-analysis was considered inappropriate and was not conducted.

### 2.5. Rating Certainty of Evidence

The certainty of evidence was graded using the Grading of Recommendations, Assessment, Development, and Evaluation (GRADE) approach for main outcomes. Primary outcomes included mortality, MV, hemodynamic support; important outcomes included coronary artery aneurysm at discharge, abnormal cardiac function, and time to defervescence as secondary outcomes.

## 3. Results

A total of 258 studies were retrieved from the database. Finally, 10 studies were selected based on the inclusion and exclusion criteria (Figure 1).

These included 2 observational studies with a control group and 8 single-arm studies. Owing to the low incidence of MIS-C, many studies included immunomodulators with pre-existing treatment and did not report the effects separately; however, the authors decided to include them as well.

Table 1 presents the characteristics of the included studies. The final 10 studies included 145 patients with MIS-C who were treated with biological immunomodulators. The median age of the 8 studies that specified the age of those treated with biological immunomodulators was 9 years, and that of the 2 studies that only mentioned total MIS-C cases was 10 years old. Anakinra (72, 49%) and infliximab (65, 44%) were used in similar proportions. Due to the heterogeneity in the sequence of treatment, a summary of the time line of therapy was included in Table S4 of Supplementary Materials.

Table 2 shows the GRADE summary of the findings from 2 studies that included a biological immunomodulator group vs. a control group treated with standard IVIg and/or corticosteroid therapy. Celikel et al. [8] included 33 severe MIS-C cases, all of whom required intensive care unit (ICU) admission and received initial immunomodulatory therapy with IVIg (1–2 g/kg) and corticosteroid as pulse methylprednisolone (control group). The study group included 23 patients who were treated with additional biological immunomodulators due to refractory response within 24 h. Anakinra was initially administered to 2 patients at a dose of 4 to 10 mg/kg per day, with a mean duration of 11 days (IQR, 6–17 days). If the patient was refractory to anakinra after 7 days, tocilizumab was administered at 8mg/kg in patients weighing more than 30 kg; this was carried out in 2 patients. The only cardiac outcome to be reported separately for the study and control group was left ventricular ejection fraction (LVEF). Upon ICU admission, patients in the group treated with immunomodulators showed lower LVEF compared to the control group (54% vs. 60%, *p* = 0.08). However, after a week of biological immunomodulatory treatment, LVEF improved from 55% to 69% (IQR 44–78, *p* < 0.001) in the study group. Other clinical outcomes were reported without distinguishing between the use of biological immunomodulators. Of the patients, 2 died, 5 were intubated, and 24 received hemodynamic support, including inotropic agents. Additionally, 2 mortality cases were not related to the use of biological immunomodulators. Abnormal cardiac function or coronary artery anomalies were observed in 18 patients (54.5%), 3 of whom had coronary ectasia [8]. Since the cardiac outcomes were not compared based on immunomodulator treatment, the major findings, aside from cardiac-related outcomes, were categorized as studies without a control group.

In another retrospective cohort study by Cole et al. [9] involving a control group, patients treated with both IVIg and infliximab (n = 52) were compared with those treated only with IVIg (n = 20). Patients in the IVIg and infliximab group, relative to the control group, initially demonstrated a higher proportion of coronary artery ectasia and/or LVEF abnormalities (40% vs. 71%, *p* = 0.03) and were admitted to the intensive care unit (ICU) (65% vs. 31%, *p* = 0.01). The rate of newly developed LVEF dysfunction or aggravation was lower (20% vs. 4%, *p* = 0.05) in the study group. Additionally, patients in the IVIg and infliximab group showed reduced C-reactive protein (CRP) levels after 24 and 48 h of treatment than those in the IVIg alone group. The time to defervescence for patients in the IVIg group was 3 days (interquartile range, IQR 2–4), whereas for patients in the IVIg and infliximab group, it was 2 days (IQR 1–3, *p* = 0.12). Further comparison of the two studies in parallel parameters are shown in Table S5 of the Supplementary Materials.

Table 3 summarizes the findings from studies without a control group. The selected studies (n = 9) were conducted on patients with MIS-C; however, those treated with biological immunomodulators were not evaluated separately in terms of clinical outcomes in some of the studies [10,11,12,16]. Celikel et al. [8] reported findings on mortality, mechanical ventilation, hemodynamic support, and cardiac abnormalities without comparing the control and study groups. Therefore this study has been presented here as a non-comparative study.

Lee et al. conducted a study including 28 patients with MIS-C, among which 5, who were unresponsive to IVIg and corticosteroid treatment, were treated with either anakinra alone (n = 1) or IVIg, methylprednisolone, and anakinra combination (n = 4) [12]. Papdopoulou et al. included 19 patients with MIS-C, of which 4 were treated with anakinra and 1 with infliximab [10]. In a study by Gruber et al. [9], 7 out of 9 patients with MIS-C were administered tocilizumab. Abdel-Haq et al. studied 33 patients with MIS-C, 12 of whom were treated with infliximab [11]. Savas Sen et al. [16], Brisca et al. [14], and Campanello et al. [13] included 9, 6, and 8 patients with MIS-C, respectively, of whom all received anakinra. Sozeri et al. examined 67 MIS-C cases, among which 17 were treated with anakinra and 1 with tocilizumab [15].

Out of the 6 studies which examined the mortality rate [8,10,11,12,15,16], 2 reported 4 cases of mortality in patients who received immunomodulators [7,15]. Regarding mechanical ventilation, 5 reports were noted [8,10,12,14,15]; however, only 2 specifically mentioned mortality cases in those who were treated with immunomodulators due to cardiac arrhythmia and sepsis unrelated to biological agent use [8,15]. Although 4 studies evaluated hemodynamic support, meta-analysis was not conducted owing to case heterogeneity [8,10,11,12]. Among the 6 studies which reported coronary artery aneurysm at discharge [2,8,10,11,12,13], only 2 clearly specified it occurred in the immunomodulator group [2,11]. In a study by Gruber et al. [2], 6 patients had coronary artery dilation or aneurysm at the time of admission, but all patients’ cardiac abnormalities eventually normalized. Abdel-Haq et al. [11] also assessed 12 patients who were treated with infliximab, among whom 4 initially had coronary artery dilatation above a Z score of 2.0 but returned to the normal range at discharge. Regarding cardiac dysfunction, 4 studies reported that the LVEF and ejection fraction decreased and recovered at discharge [10,11,12,13]. Clinical improvement in 1 study reported a median duration of 6 days [2]. Time to defervescence was observed in 2 studies, in which the median durations were 4 and 10 days respectively [10,12]. However, cardiac abnormalities, clinical improvement, and defervescence have not been specifically examined in patients receiving biological immunomodulators.

## 4. Discussion

This systematic review was based on 10 studies, including 145 patients with refractory MIS-C who received biological immunomodulators. Most studies used anakinra and infliximab with a small proportion using tocilizumab. Positive clinical outcomes, such as cardiac complications, time to defervescence, and inflammatory marker normalization, have been observed with biological immunomodulator treatment. However, due to the heterogeneity in sample and treatment, interpretation is limited and the level of evidence can, therefore, be described as “very low”. Nevertheless, available evidence indicates a beneficial role for biological immunomodulators in treating patients with refractory MIS-C who do not respond to first-line IVIg and/or corticosteroid treatment. Moreover, consultation with pediatric rheumatologists and infectious disease specialists to make clinical judgments on a case-by-case basis is recommended.

### 4.1. Implications

The studies included in this review are meaningful because they reflect real-world clinical practice in treating rare refractory MIS-C cases. As recommended, most institutions used IVIg and/or corticosteroid as first-line therapy, and only one study has reported a case that was initially treated with a biological immunomodulator. Until this systematic review was conducted, there were no results from randomized controlled trials; therefore, only two case-control studies were included, while the rest were case series. Among the case-control studies, one used anakinra and the other used infliximab. Furthermore, no case-control studies using tocilizumab have been reported; only one case series has been noted. Thus, the effectiveness of immunomodulators as a first-line therapy for MIS-C is unclear. However, studies suggest that when a patient persistently shows signs of systemic inflammation and cardiac dysfunction after initial IVIg and corticosteroid treatment, biologics as second-line therapy or in conjunction with IVIg may benefit the clinical course. It is noteworthy that only one study, so far, has examined the adverse effects related to immunomodulatory treatment, and 12 patients treated with infliximab did not report any adverse effects related to the drug or infectious complications. So far, it is not known to have any deleterious adverse effect; however, future studies should continue to examine the safety issue of the biologic immunomodulators.

### 4.2. Comparison to Pre-Existing Evidence and Guidelines

These findings are consistent with pre-existing guidelines stating that biological immunomodulators may be considered in patients with refractory MIS-C. For instance, the American College of Rheumatology, updated in 2022, recommends that anakinra or infliximab should be considered because of reported positive outcomes [4]. However, it can be concluded that anakinra may be a better option than infliximab, as more studies support its beneficial role. The American Academy of Pediatrics also mentions anakinra as the only biological immunomodulatory agent considered for treating refractory MIS-C [5]. In another guideline established in Australia, refractory MIS-C can be managed by increasing the dose of glucocorticoids, using a second dose of high-dose IVIg, or by adding biological immunomodulators, such as anti-IL-1, anti-IL-6, or anti-TNF agents [6]. The studies included in this review support the current recommendation that all three classes of biological immunomodulators can be considered as second-line treatments, but anakinra is the most commonly used one and has accumulated more clinical evidence.

### 4.3. Considerations Regarding Choice of Biologic Agents

Owing to the diverse options of immunomodulators to treat MIS-C, several considerations have to be made during selection. Although anakinra has wide applicability in treating systemic juvenile idiopathic arthritis, cryopyrin-related periodic syndrome, and macrophage activation syndrome [17], and has also been reported to be effective in the treatment of Kawasaki disease, its use can be limited in terms of cost and drug accessibility. Anakinra is available in several countries. For instance, in Korea, it can only be obtained from the Korea Orphan and Essential Drug Center and is not covered by national health insurance. Infliximab is recommended by the National Health Insurance for the treatment of refractory Kawasaki disease and IBD. However, because infliximab is contraindicated in patients with macrophage activation syndrome (MAS), patients with MIS-C showing similar signs should not be treated with this immunomodulator. Lastly, although tocilizumab has been shown to be effective in systemic and polyarticular juvenile idiopathic arthritis and MAS, only a limited number of studies have evaluated its use in MIS-C; therefore, its risks and benefits are still unclear.

### 4.4. Limitations

The limitations of this review include its heterogeneous design and lack of parallel parameters between control and intervention group in the included studies. Due to the rarity of MIS-C cases and diverse number of biologic agents as options, it is difficult to conduct prospective studies with control groups analyzing each immunomodulator and to have an adequate sample size, which limits the interpretation. However, the inclusion of studies that encompassed a large number of samples worldwide was attempted. Therefore, future studies should aim to investigate each biologic agent separately and also conduct randomized controlled studies with a larger sample size.

## 5. Conclusions

This systematic review suggests the potential of biological immunomodulators for the timely treatment of severe MIS-C refractory to first-line IVIg and corticosteroid treatments. As a widely accepted consensus on the treatment of MIS-C has not been attained, the choice of biological immunomodulators should be carefully made on an individual basis by pediatric rheumatologists and infectious disease specialists to assess their risks and benefits. Furthermore, monitoring adverse effects and infectious complications is necessary. To provide reliable guidelines for the use of biological immunomodulators in the treatment of MIS-C, well-designed randomized controlled trials should be conducted in the future.

## Figures and Tables

**Table 1:** Summary of the selected studies.

Source	Design	Study Period	Cases Using Biologics/Total MIS-C Cases (%)	Median Age of Patients Treated with Biologics (Range) /No. of Cases < 10 Years Old (%)	Biologics Used	ICU Admission Cases (%)
Celikel et al. [8], 2021	Case-control	2020.9–2020.10	23/33 (70)	12.0 (3–17)/ 17 (52)	Anakinra	33 (100)
Cole et al. [9], 2021	Retrospective observational	2020.4–2021.2	52/72 (72)	9.0 (6–12)/ N/A	Infliximab	42 (58)
Gruber et al. [2], 2020	Case series	2020.4–2020.6	7/9 (78)	12.0 ^b^/^c^	Tocilizumab	N/A
Papadopoulou et al. [10], 2021	Case series	2020.4–2020.5	5/19 (26)	13.9 (7.1–14.4)/ 10 (53)	Anakinra (4), Infliximab (1)	17 (90)
Abdel-Haq et al. [11], 2021	Case series	2020.4–2020.6	12/33 (36)	8.0 (IQR 7–14)/ 28 (85)	Infliximab	22 (67)
Lee et al. [12], 2020	Case series	2020.3–2020.6	5/28 (18)	9.0 (0.1–17)/ ^d^	Anakinra	17 (61)
Campanello et al. [13], 2022	Retrospective observational	2020.3–2021.9	8/25 (32)	5.0 (IQR 3–12) ^a^/^e^	Anakinra	N/A
Brisca et al. [14], 2021	Retrospective observational	2020.4–2021.6	6/23 (26)	5.8 (4–12) ^a^/N/A	Anakinra	0 (0)
Sozeri et al. [15], 2021	Prospective observational	2020.4–2021.4	18/67 (27)	11.2 ^b^/N/A	Anakinra (17), Tocilizumab (1)	21 (31)
Savas Sen et al. [16], 2020	Case series	2020.8–2021.3	9/45 (20)	14.0 (IQR 12–14)/ ^f^	Anakinra (9)	11 (24)

Abbreviations: IQR, interquartile range. N/A, information not available. ^a^ Median age of patients in the group treated with biologics was not specified; therefore, the age of all MIS-C cases who received diverse treatment was noted. ^b^ IQR was not mentioned nor accessible. ^c^ This study categorized patients into its own age groups: 2 patients (22%) were included in the 0–6-year-old group, 5 (56%) in the 7–13-year-old group, and 2 (22%) in the 14–20-year-old group. ^d^ Ages of all the patients were not shown; however, the ages of those with coronary artery abnormalities were available. Out of 7 patients, 5 (71%) were less than 10 years old, ^e^ 13 patients (52%) were less than 6 years old, and 12 (48%) were more than 6 years old. ^f^ The ages of the patients admitted to ICU and administered biologic immunomodulator were available; 2 patients (33%) were less than 10 years old, while 4 (66%) were more than 10 years old.

**Table 2:** Studies with a control group.

Outcomes ^a^	Summary	No. of Participants (Studies)	Certainty of the Evidence (GRADE)
Mortality	Not reported		
Mechanical ventilation	Not reported		
Hemodynamic support	Not reported		
Coronary aneurysm at discharge	Not reported		
Cardiac dysfunction	1. Celikel et al.: Decreased cardiac function in biological immunomodulator group vs. control group (*p* = 0.08) [8]. 2. Cole et al.: Worsened left ventricular systolic function in 4/52 cases with IVIg + infliximab compared to 5/20 cases with IVIg alone [9].	102 (2 observational studies)	⊕◯◯◯ Very low ^b,c^ (important)
Fever	1. Cole et al.: Time to defervescence was 3 days (IQR 2–4) for IVIg alone vs. 2 days (IQR 1–3) for IVIg + infliximab [9].	72 (1 observational study)	⊕◯◯◯ Very low ^c^ (important)

Abbreviations: IVIg, intravenous immunoglobulin. ^a^ Celikel et al. reported the results based on these parameters; however, they were not defined according to the treatment group. Therefore, major outcomes excluding cardiac dysfunction were classified as studies without a comparison group and analyzed. ^b^ The settings of the immunomodulator group and control group are unclear. ^c^ Small sample size. GRADE Working Group grades of evidence. High certainty: very high confidence that the true effect lies close to that of the estimate of the effect. Moderate certainty: moderate confidence in the effect estimate: the true effect is likely to be close to the estimate of the effect, but there is a possibility that it is substantially different. Low certainty: confidence in the effect estimate is limited: the true effect may be substantially different from the estimate of the effect. Very low certainty: very little confidence in the effect estimate: the true effect is likely to be substantially different from the estimate of effect.

**Table 3:** Studies without a control group.

Outcomes	Summary	No of Participants (Studies)	Certainty of the Evidence (GRADE)
Mortality	Reported in 6 studies. Celikel et al.: 2 patients (6%) died; 1 received IVIg, glucocorticoid, anakinra, and plasmapheresis and died of severe sepsis [8]. Sozeri et al.: 2 cases of mortality (3%) [15].	100 (2 observational study)	⊕◯◯◯ Very low ^a^ (Critical)
Mechanical ventilation	Reported in 5 studies. Outcomes in patients treated with biological immunomodulators: Brisca et al.: No cases required mechanical ventilation [14]. Sozeri et al.: 2 cases (3%) required mechanical ventilation; 2 cases (3%) received ECMO support [15]. Outcomes reported without distinguishing between biological immunomodulator-treated groups and other drugs: Çelikel et al.: 5 patients (15%) were intubated [8]. Lee et al.: 1 patient (4%) was mechanically ventilated; patient underwent tracheostomy upon admission [12]. Papadopoulou et al.: 10 patients (53%) were mechanically ventilated; median duration was 3 days (IQR 1–4) [10].	92 (5 observational studies)	⊕◯◯◯ Very low ^a^ (Critical)
Hemodynamic support	Reported in 4 studies. Çelikel et al.: Majority of patients (24, 73%) required vasoactive drugs [8]. Abdel-Haq et al.: 9 patients (75%) needed vasopressors [11]. Lee et al.: 7 patients (25%) used inotropes; none required ECMO support [12]. Papadopoulou et al.: Seventeen patients (89%) used noradrenaline and milrinone for hemodynamic support [10].	92 (4 observational studies)	⊕◯◯◯ Very low ^a^ (Critical)
Coronary aneurysm at discharge	Reported in 6 studies. Studies separately reported the clinical outcomes in patient group treated with biological immunomodulators: Gruber et al.: 6 patients (67%) had artery ectasia or aneurysm at admission, which normalized at discharge [2]. Abdel-Haq et al.: 4 patients (33%) treated with infliximab had coronary artery ectasia at admission, which normalized at discharge [11].	164 (6 observational studies)	⊕◯◯◯ Very low ^a^ (important)
Cardiac dysfunction	Reported in 4 studies. Findings from studies reporting on biological immunomodulator-treated patients: Abdel-Haq et al.: 8 patients (67%) had an ejection fraction (EF) lower than 55% [11]. Findings from studies not differentiating between biological immunomodulator-treated patients: Campanello et al.: 10 patients (40%) had QT abnormalities; 1 patient’s condition did not normalize [13]. Lee et al.: Eleven patients (39%) had EF < 55% [12]. Papadopoulou et al.: 1 patient (5%) had left ventricular dysfunction and myocarditis; received ECMO support [10].	84 (4 observational studies)	⊕◯◯◯ Very low ^a^ (important)
Clinical improvement	Gruber et al.: Median length of hospitalization was 6 days [2].	9 (1 observational study)	⊕◯◯◯ Very low ^a^ (important)
Fever	Reported in 2 studies. Lee et al.: Time to defervescence was 2.5 days [12]. Papadopoulou et al.: Median time to defervescence and CRP normalization was 10 days (IQR 7.8–11) [10].	47 (2 observational studies)	⊕◯◯◯ Very low ^a^ (important)

Abbreviations: IQR, interquartile range. ^a^ Small sample size. GRADE Working Group grades of evidence. High certainty: very high confidence that the true effect lies close to that of the estimate of the effect. Moderate certainty: moderately confidence in the effect estimate: the true effect is likely to be close to the estimate of the effect, but there is a possibility that it is substantially different. Low certainty: confidence in the effect estimate is limited: the true effect may be substantially different from the estimate of the effect. Very low certainty: very little confidence in the effect estimate: the true effect is likely to be substantially different from the estimate of effect.

## Data Availability

The original contributions presented in the study are included in the article, further inquiries can be directed to the corresponding author. A preprint has previously been published [18].

## Supplementary Materials

The following supporting information can be downloaded at: https://www.mdpi.com/article/10.3390/children11101180/s1. Table S1. PRISMA checklist; Table S2. Search terms and strategy; Figure S1. Risk of bias graph; Table S3. Risk of bias summary; Table S4. Summary of sequence of immunomodulatory treatment; Table S5. Comparison of Cole et al. and Celikel et al. studies.
