# Supplementary material for: Clinical Effectiveness of Biological Immunomodulators in SARS-CoV-2-Associated Multisystem Inflammatory Syndrome in Children: A Systematic Review
Source: Children (Basel). 2024 Sep 27;11(10):1180. doi: 10.3390/children11101180 (PMC11506526; doi:10.3390/children11101180)
Supplement: Supplementary file 1 [file children-11-01180-s001.zip › children-3164323-supplementary.pdf]

Supplementary material

Table S1. PRISMA checklist

Table S2. Search terms and strategy

1) **PubMED**
2) **OVID-EMBASE**
3) **Cochrane library CENTRAL**
4) **KMBASE**

Figure S1. Risk of bias graph

Table S3. Risk of bias summary

Table S4. Summary of sequence of immunomodulatory treatment

Table S5. Comparison of Cole et al. and Celikel et al. studies

This supplementary material has been provided by the authors to give readers additional information about their work.

Table S1. PRISMA checklist

Section/Topic	Item #	Checklist item	Location
TITLE			
Title	1	Identify the report as a systematic review.	1
ABSTRACT			
Abstract	2	See the PRISMA 2020 for Abstracts checklist.	3
INTRODUCTION			
Rationale	3	Describe the rationale for the review in the context of existing knowledge.	5-6
Objectives	4	Provide an explicit statement of the objective(s) or question(s) the review addresses.	7
METHODS			
Eligibility criteria	5	Specify the inclusion and exclusion criteria for the review and how studies were grouped for the syntheses.	8
Information sources	6	Specify all databases, registers, websites, organisations, reference lists and other sources searched or consulted to identify studies. Specify the date when each source was last searched or consulted.	8
Search strategy	7	Present the full search strategies for all databases, registers and websites, including any filters and limits used.	eTable 2 of Supplementary material
Selection process	8	Specify the methods used to decide whether a study met the inclusion criteria of the review, including how many reviewers screened each record and each report retrieved, whether they worked independently, and if applicable, details of automation tools used in the process.	9-10
Data collection process	9	Specify the methods used to collect data from reports, including how many reviewers collected data from each report, whether they worked independently, any processes for obtaining or confirming data from study investigators, and if applicable, details of automation tools used in the process.	9-10
Data items	10a	List and define all outcomes for which data were sought. Specify whether all results that were compatible with each outcome domain in each study were sought (e.g. for all measures, time points, analyses), and if not, the methods used to decide which results to collect.	10
	10b	List and define all other variables for which data were sought (e.g. participant and intervention characteristics, funding sources). Describe any assumptions made about any missing or unclear information.	8-9
Study risk of bias assessment	11	Specify the methods used to assess risk of bias in the included studies, including details of the tool(s) used, how many reviewers assessed each study and whether they worked independently, and if applicable, details of automation tools used in the process.	9
Effect measures	12	Specify for each outcome the effect measure(s) (e.g. risk ratio, mean difference) used in the synthesis or presentation of results.	10
Synthesis methods	13a	Describe the processes used to decide which studies were eligible for each synthesis (e.g. tabulating the study intervention characteristics and comparing against the planned groups for each synthesis (item #5)).	8,
	13b	Describe any methods required to prepare the data for presentation or synthesis, such as handling of missing summary statistics, or data conversions.	9

Section/Topic	Item #	Checklist item	Location
	13c	Describe any methods used to tabulate or visually display results of individual studies and syntheses.	9
	13d	Describe any methods used to synthesize results and provide a rationale for the choice(s). If meta-analysis was performed, describe the model(s), method(s) to identify the presence and extent of statistical heterogeneity, and software package(s) used.	9
	13e	Describe any methods used to explore possible causes of heterogeneity among study results (e.g. subgroup analysis, meta-regression).	9
	13f	Describe any sensitivity analyses conducted to assess robustness of the synthesized results.	Not applicable
Reporting bias assessment	14	Describe any methods used to assess risk of bias due to missing results in a synthesis (arising from reporting biases).	Not applicable
Certainty assessment	15	Describe any methods used to assess certainty (or confidence) in the body of evidence for an outcome.	10
RESULTS			
Study selection	16a	Describe the results of the search and selection process, from the number of records identified in the search to the number of studies included in the review, ideally using a flow diagram.	11, Figure 1
	16b	Cite studies that might appear to meet the inclusion criteria, but which were excluded, and explain why they were excluded.	Not applicable
Study characteristics	17	Cite each included study and present its characteristics.	Table 1
Risk of bias in studies	18	Present assessments of risk of bias for each included study.	9, eTable 3
Results of individual studies	19	For all outcomes, present, for each study: (a) summary statistics for each group (where appropriate) and (b) an effect estimate and its precision (e.g. confidence/credible interval), ideally using structured tables or plots.	11-13
Results of syntheses	20a	For each synthesis, briefly summarise the characteristics and risk of bias among contributing studies.	eTable3
	20b	Present results of all statistical syntheses conducted. If meta-analysis was done, present for each the summary estimate and its precision (e.g. confidence/credible interval) and measures of statistical heterogeneity. If comparing groups, describe the direction of the effect.	11-14, Table 2-3
	20c	Present results of all investigations of possible causes of heterogeneity among study results.	8-9,11
	20d	Present results of all sensitivity analyses conducted to assess the robustness of the synthesized results.	Not applicable
Reporting biases	21	Present assessments of risk of bias due to missing results (arising from reporting biases) for each synthesis	eTable 3

Section/Topic	Item #	Checklist item	Location
		assessed.	
Certainty of evidence	22	Present assessments of certainty (or confidence) in the body of evidence for each outcome assessed.	Table 2-3
DISCUSSION			
Discussion	23a	Provide a general interpretation of the results in the context of other evidence.	15
	23b	Discuss any limitations of the evidence included in the review.	17
	23c	Discuss any limitations of the review processes used.	17
	23d	Discuss implications of the results for practice, policy, and future research.	16-18
OTHER INFORMATION			
Registration and protocol	24a	Provide registration information for the review, including register name and registration number, or state that the review was not registered.	CRD42023417146
	24b	Indicate where the review protocol can be accessed, or state that a protocol was not prepared.	https://www.crd.york.ac.uk/PROSPERO.
	24c	Describe and explain any amendments to information provided at registration or in the protocol.	Not applicable
Support	25	Describe sources of financial or non-financial support for the review, and the role of the funders or sponsors in the review.	18
Competing interests	26	Declare any competing interests of review authors.	18
Availability of data, code and other materials	27	Report which of the following are publicly available and where they can be found: template data collection forms; data extracted from included studies; data used for all analyses; analytic code; any other materials used in the review.	25-26 CRD42023417146

Table S2. Search terms and strategy

1) **PubMED**

no.	Search strategy
#1	"COVID-19"[Mesh]
#2	"COVID-19"[TW] OR "COVID 19"[TW] OR "COVID-19 Virus Disease"[TW] OR "COVID 19 Virus Disease"[TW] OR "COVID-19 Virus Diseases"[TW] OR "Disease, COVID-19 Virus"[TW] OR "Virus Disease, COVID-19"[TW] OR "COVID-19 Virus Infection"[TW] OR "COVID 19 Virus Infection"[TW] OR "COVID-19 Virus Infections"[TW] OR "Infection, COVID-19 Virus"[TW] OR "Virus Infection, COVID-19"[TW] OR "2019-nCoV Infection"[TW] OR "2019 nCoV Infection"[TW] OR "2019-nCoV Infections"[TW] OR "Infection, 2019-nCoV"[TW] OR "Coronavirus Disease-19"[TW] OR "Coronavirus Disease 19"[TW] OR "2019-nCoV Disease"[TW] OR "2019 nCoV Disease"[TW] OR "2019-nCoV Diseases"[TW] OR "Disease, 2019-nCoV"[TW] OR "COVID19"[TW] OR "Coronavirus Disease 2019"[TW] OR "Disease 2019, Coronavirus"[TW] OR "SARS Coronavirus 2 Infection"[TW] OR "SARS-CoV-2 Infection"[TW] OR "Infection, SARS-CoV-2"[TW] OR "SARS CoV 2 Infection"[TW] OR "SARS-CoV-2 Infections"[TW] OR "COVID-19 Pandemic"[TW] OR "COVID 19 Pandemic"[TW] OR "COVID-19 Pandemics"[TW] OR "Pandemic, COVID-19"[TW] OR "2019 Novel Coronavirus Disease"[TW] OR "2019 Novel Coronavirus Infection"[TW] OR "coronavirus disease 2"[TW] OR "coronavirus disease 2019 pneumonia"[TW] OR "coronavirus infection 2019"[TW] OR "COVID"[TW] OR "COVID 19 induced pneumonia"[TW] OR "COVID 2019"[TW] OR "COVID-19 induced pneumonia"[TW] OR "COVID-19 pneumonia"[TW] OR "nCoV 2019 disease"[TW] OR "nCoV 2019 infection"[TW] OR "paucisymptomatic coronavirus disease 2019"[TW] OR "SARS coronavirus 2 pneumonia"[TW] OR "SARSCoV2 disease"[TW] OR "SARS-CoV2 disease"[TW] OR "SARS-CoV-2 disease"[TW] OR "SARSCoV2 infection"[TW] OR "SARS-CoV2 infection"[TW] OR "SARS-CoV-2 pneumonia"[TW] OR "severe acute respiratory syndrome 2"[TW] OR "severe acute respiratory syndrome 2 pneumonia"[TW] OR "severe acute respiratory syndrome coronavirus 2 infection"[TW] OR "severe acute respiratory syndrome coronavirus 2019 infection"[TW] OR "severe acute respiratory syndrome CoV-2 infection"[TW] OR "Wuhan coronavirus disease"[TW] OR "Wuhan coronavirus infection"[TW] OR "2019 novel coronavirus epidemic"[TW] OR "new coronavirus pneumonia"[TW] OR "novel coronavirus 2019 disease"[TW] OR "novel coronavirus 2019 infection"[TW] OR "novel coronavirus disease 2019"[TW] OR "novel coronavirus infected pneumonia"[TW] OR "novel coronavirus infection 2019"[TW] OR "novel coronavirus pneumonia"[TW] OR "2019nCoV"[TW] OR "19nCoV"[TW] OR "COVID19*"[TW] OR "SARSCOV-2"[TW] OR "SARSCOV2"[TW] OR "corona virus 2"[TW] OR "Wuhan"[TW] OR "Hubei"[TW] OR "new coronavirus"[TW] OR "novel coronavirus"[TW] OR "novel corona virus"[TW] OR "novel CoV"[TW]
#3	"SARS-CoV-2"[Mesh]

#4	"SARS-CoV-2"[TW] OR "Coronavirus Disease 2019 Virus"[TW] OR "Wuhan Seafood Market Pneumonia Virus"[TW] OR "SARS-CoV-2 Virus"[TW] OR "SARS CoV 2 Virus"[TW] OR "SARS-CoV-2 Viruses"[TW] OR "Virus, SARS-CoV-2"[TW] OR "2019-nCoV"[TW] OR "COVID-19 Virus"[TW] OR "COVID 19 Virus"[TW] OR "COVID-19 Viruses"[TW] OR "Virus, COVID-19"[TW] OR "Wuhan Coronavirus"[TW] OR "Coronavirus, Wuhan"[TW] OR "SARS Coronavirus 2"[TW] OR "Coronavirus 2, SARS"[TW] OR "Severe Acute Respiratory Syndrome Coronavirus 2"[TW] OR "2019 Novel Coronavirus"[TW] OR "2019 Novel Coronaviruses"[TW] OR "Coronavirus, 2019 Novel"[TW] OR "Novel Coronavirus, 2019"[TW] OR "2019 nCOV"[TW] OR "2019 severe acute respiratory syndrome coronavirus 2"[TW] OR "HCoV-19"[TW] OR "Human coronavirus 2019"[TW] OR "nCoV-2019"[TW] OR "SARS2 (virus)"[TW] OR "SARS-related coronavirus 2"[TW] OR "Sever acute respiratory syndrome coronavirus 2"[TW] OR "Severe acute respiratory coronavirus 2"[TW] OR "Severe acute respiratory syndorme coronavirus 2"[TW] OR "severe acute respiratory syndrome 2 virus"[TW] OR "severe acute respiratory syndrome corona virus 2"[TW] OR "severe acute respiratory syndrome coronavirus 2019"[TW] OR "Severe acute respiratory syndrome coronoavirus 2"[TW] OR "Severe acute respiratory syndrome coronvirus 2"[TW] OR "severe acute respiratory syndrome CoV-2 virus"[TW] OR "Severe acute respiratory syndrome related coronavirus 2"[TW] OR "Severe acute respiratory syndrome virus 2"[TW] OR "Severe acute respiratoy syndrome coronavirus 2"[TW] OR "2019 new coronavirus"[TW] OR "novel 2019 coronavirus"[TW] OR "novel coronavirus-19"[TW]
#5	#1 OR #2 OR #3 OR #4
#6	"pediatric multisystem inflammatory disease, COVID-19 related" [Supplementary Concept]

#7	"pediatric multisystem inflammatory disease, COVID-19 related"[TW] OR "pediatric multisystem inflammatory syndrome, SARS-CoV-2 related"[TW] OR "pediatric multisystem inflammatory syndrome, COVID-19 related"[TW] OR "pediatric multi-system inflammatory syndrome, COVID-19 related"[TW] OR "pediatric multi-system inflammatory syndrome, SARS-CoV-2 related"[TW] OR "MISC associated with COVID-19"[TW] OR "multisystem inflammatory syndrome, pediatric, COVID-19 related"[TW] OR "multi-system inflammatory disease, pediatric, COVID-19 related"[TW] OR "multi-system inflammatory syndrome, pediatric, COVID-19 related"[TW] OR "PIMS-TS"[TW] OR "multisystem inflammatory syndrome in children MIS-C associated with COVID-19"[TW] OR "MIS-C associated with COVID-19"[TW] OR "pediatric multi-system inflammatory disease, COVID-19 related"[TW] OR "multisystem inflammatory disease, pediatric, COVID-19 related"[TW] OR "pediatric inflammatory multisystem syndrome"[TW] OR "MIS-C multisystem inflammatory syndrome in children"[TW] OR "multisystem inflammatory syndrome in children"[TW] OR "pediatric multisystem inflammatory syndrome"[TW] OR "childhood multisystem inflammatory syndrome"[TW] OR "COVID-19 associated Kawasaki-like multisystem inflammatory disease"[TW] OR "COVID-19 associated multisystem inflammatory syndrome"[TW] OR "COVID-19 Kawasaki-like syndrome"[TW] OR "COVID-19 related multisystem inflammatory syndrome"[TW] OR "Kawa-COVID-19"[TW] OR "Kawasaki like disease"[TW] OR "Kawasaki-like multisystem inflammatory syndrome"[TW] OR "MIS-C (multisystem inflammatory syndrome in children)"[TW] OR "multi-inflammatory syndrome in children"[TW] OR "multiorgan inflammatory syndrome associated with SARS-CoV-2"[TW] OR "multisystem inflammation syndrome in children"[TW] OR "multisystem inflammatory syndrome associated with SARS-CoV-2"[TW] OR "multi-system inflammatory syndrome in children"[TW] OR "paediatric inflammatory multisystem syndrome temporally associated with severe acute respiratory syndrome coronavirus 2"[TW] OR "paediatric multisystem inflammatory syndrome"[TW] OR "paediatric multi-system inflammatory syndrome"[TW] OR "paediatric multisystem inflammatory syndrome temporally associated with COVID-19"[TW] OR "paediatric multisystem inflammatory syndrome temporally associated with SARS-CoV-2"[TW] OR "pediatric inflammatory multisystem syndrome temporally associated with SARS-CoV-2"[TW] OR "pediatric inflammatory multisystem syndrome temporally associated with severe acute respiratory syndrome coronavirus 2"[TW] OR "pediatric multi-inflammatory syndrome"[TW] OR "pediatric multi-inflammatory syndrome mimicking Kawasaki disease"[TW] OR "pediatric multisystem inflammatory disease"[TW] OR "pediatric multi-system inflammatory syndrome"[TW] OR "pediatric multisystem inflammatory syndrome associated with COVID-19"[TW] OR "pediatric multisystem inflammatory syndrome temporally associated with COVID-19"[TW] OR "pediatric multisystem inflammatory syndrome temporally associated with SARS-CoV-2"[TW] OR "PMIS-TS"[TW] OR "SARS-CoV-2 associated multisystem inflammatory syndrome"[TW] OR "SARS-CoV-2 induced Kawasaki-like hyperinflammatory syndrome"[TW] OR "SARS-CoV-2 mimicking Kawasaki disease"[TW] OR "pediatric multi-system inflammatory disease"[TW] OR "MISC"[TW] OR "MIS-C"[TW] OR "multisystem inflammatory syndrome"[TW] OR "multi-system inflammatory syndrome"[TW] OR "multisystem inflammatory disease"[TW] OR "multi-system inflammatory disease"[TW]
#8	#6 OR #7
#9	#5 AND #8
#10	"Interleukin 1 Receptor Antagonist Protein"[Mesh]
#11	"Interleukin 1 Receptor Antagonist Protein"[TW] OR "IL1 Febrile Inhibitor"[TW] OR "Febrile Inhibitor, IL1"[TW] OR "IL-1Ra"[TW] OR "Urine-Derived IL1 Inhibitor"[TW] OR "IL1 Inhibitor, Urine-Derived"[TW] OR "Urine Derived IL1 Inhibitor"[TW] OR "IL-1 Inhibitor, Urine"[TW] OR "IL 1 Inhibitor, Urine"[TW] OR "Urine IL-1 Inhibitor"[TW] OR "Interleukin 1 Inhibitor, Urine"[TW] OR "Antril"[TW] OR "Kineret"[TW] OR "Anakinra"[TW] OR "recombinant interleukin 1 receptor antagonist"[TW] OR "recombinant interleukin 1 receptor blocker"[TW] OR "recombinant interleukin 1 receptor blocking agent"[TW]

#12	interleukin-1 inhibitor*[TW] OR IL-1 inhibitor*[TW] OR "interleukin-1 inhibitor"[TW] OR "IL-1 inhibitor"[TW] OR "interleukin-1 receptor antagonist"[TW] OR "IL-1 receptor antagonist"[TW]
#13	"Infliximab"[Mesh]
#14	"Infliximab"[TW] OR "Monoclonal Antibody cA2"[TW] OR "cA2, Monoclonal Antibody"[TW] OR "MAb cA2"[TW] OR "Infliximab-abda"[TW] OR "Renflexis"[TW] OR "Infliximab-dyyb"[TW] OR "Inflectra"[TW] OR "Remicade"[TW] OR "abp 710"[TW] OR "abp710"[TW] OR "avakine"[TW] OR "avsola"[TW] OR "flixabi"[TW] OR "gp 1111"[TW] OR "gp1111"[TW] OR "inflectra"[TW] OR "infliximab abda"[TW] OR "infliximab axxq"[TW] OR "infliximab dyyb"[TW] OR "infliximab qbtx"[TW] OR "infliximab-abda"[TW] OR "infliximab-axxq"[TW] OR "infliximab-dyyb"[TW] OR "infliximab-qbtx"[TW] OR "ixifi"[TW] OR "pf 06438179"[TW] OR "pf 6438179"[TW] OR "pf06438179"[TW] OR "pf6438179"[TW] OR "remsima"[TW] OR "renflexis"[TW] OR "revellex"[TW] OR "ta 650"[TW] OR "ta650"[TW] OR "zessly"[TW]
#15	"tocilizumab"[Supplementary Concept]
#16	"Tocilizumab"[TW] OR "RHPM-1"[TW] OR "RG-1569"[TW] OR "R-1569"[TW] OR "MSB11456"[TW] OR "MSB-11456"[TW] OR "atlizumab"[TW] OR "monoclonal antibody, MRA"[TW] OR "RO-4877533"[TW] OR "Actemra"[TW] OR "Roactemra"[TW] OR "actemra 200"[TW] OR "lusinex"[TW] OR "r 1569"[TW] OR "r1569"[TW]
#17	interleukin-6 inhibitor*[TW] OR IL-6 inhibitor*[TW] OR "interleukin-6 inhibitor"[TW] OR "IL-6 inhibitor"[TW] OR "interleukin-6 receptor antagonist"[TW] OR "IL-6 receptor antagonist"[TW]
#18	"COVID-19 serotherapy" [Supplementary Concept]
#19	"COVID-19 serotherapy"[TW] OR "COVID19 serum therapy"[TW] OR "coronavirus disease-19 serotherapy"[TW] OR "COVID19 serotherapy"[TW] OR "coronavirus disease 2019 serotherapy"[TW] OR "COVID-19 serum therapy"[TW] OR "COVID-19 convalescent serum treatment"[TW] OR "SARS-CoV-2 convalescent sera treatment"[TW] OR "SARS-CoV-2 convalescent serum treatment"[TW] OR "Covid-19 convalescent sera treatment"[TW] OR "convalescent serum treatment for Covid-19"[TW] OR "COVID-19 hyperimmune globulin therapy"[TW] OR "COVID19 hyperimmune globulin therapy"[TW] OR "hyperimmune globulin therapy for COVID-19"[TW] OR "COVID-19 convalescent plasma treatment"[TW] OR "convalescent plasma treatment for Covid-19"[TW] OR "SARS-CoV-2 convalescent plasma treatment"[TW] OR "convalescent plasma"[TW] OR "convalescence phase plasma"[TW] OR "convalescence plasma"[TW] OR "convalescent human plasma"[TW] OR "convalescent immune plasma"[TW] OR "convalescent patient plasma"[TW] OR "convalescent phase plasma"[TW] OR "plasma from convalescent"[TW] OR "plasma from convalescents"[TW] OR "PlasmAr"[TW]
#20	#10 OR #11 OR #12 OR #13 OR #14 OR #15 OR #16 OR #17 OR #18 OR #19
#21	#9 AND #20
#22	#21 AND (2020/3/1:2021/12/31[pdat])

2) OVID-EMBASE

no.	Search strategy
#1	exp coronavirus disease 2019/
#2	("COVID-19" OR "COVID 19" OR "COVID-19 Virus Disease" OR "COVID 19 Virus Disease" OR "COVID-19 Virus Diseases" OR "Disease, COVID-19 Virus" OR "Virus Disease, COVID-19" OR "COVID-19 Virus Infection" OR "COVID 19 Virus Infection" OR "COVID-19 Virus Infections" OR "Infection, COVID-19 Virus" OR "Virus Infection, COVID-19" OR "2019-nCoV Infection" OR "2019 nCoV Infection" OR "2019-nCoV Infections" OR "Infection, 2019-nCoV" OR "Coronavirus Disease-19" OR "Coronavirus Disease 19" OR "2019-nCoV Disease" OR "2019 nCoV Disease" OR "2019-nCoV Diseases" OR "Disease, 2019-nCoV" OR "COVID19" OR "Coronavirus Disease 2019" OR "Disease 2019, Coronavirus" OR "SARS Coronavirus 2 Infection" OR "SARS-CoV-2 Infection" OR "Infection, SARS-CoV-2" OR "SARS CoV 2 Infection" OR "SARS-CoV-2 Infections" OR "COVID-19 Pandemic" OR "COVID 19 Pandemic" OR "COVID-19 Pandemics" OR "Pandemic, COVID-19" OR "2019 Novel Coronavirus Disease" OR "2019 Novel Coronavirus Infection" OR "coronavirus disease 2" OR "coronavirus disease 2019 pneumonia" OR "coronavirus infection 2019" OR "COVID" OR "COVID 19 induced pneumonia" OR "COVID 2019" OR "COVID-19 induced pneumonia" OR "COVID-19 pneumonia" OR "nCoV 2019 disease" OR "nCoV 2019 infection" OR "paucisymptomatic coronavirus disease 2019" OR "SARS coronavirus 2 pneumonia" OR "SARSCoV2 disease" OR "SARS-CoV2 disease" OR "SARS-CoV-2 disease" OR "SARSCoV2 infection" OR "SARS-CoV2 infection" OR "SARS-CoV-2 pneumonia" OR "severe acute respiratory syndrome 2" OR "severe acute respiratory syndrome 2 pneumonia" OR "severe acute respiratory syndrome coronavirus 2 infection" OR "severe acute respiratory syndrome coronavirus 2019 infection" OR "severe acute respiratory syndrome CoV-2 infection" OR "Wuhan coronavirus disease" OR "Wuhan coronavirus infection" OR "2019 novel coronavirus epidemic" OR "new coronavirus pneumonia" OR "novel coronavirus 2019 disease" OR "novel coronavirus 2019 infection" OR "novel coronavirus disease 2019" OR "novel coronavirus infected pneumonia" OR "novel coronavirus infection 2019" OR "novel coronavirus pneumonia" OR "2019nCoV" OR "19nCoV" OR "COVID19*" OR "SARSCOV-2" OR "SARSCOV2" OR "corona virus 2" OR "Wuhan" OR "Hubei" OR "new coronavirus" OR "novel coronavirus" OR "novel corona virus" OR "novel CoV").mp.
#3	exp Severe acute respiratory syndrome coronavirus 2/
#4	("SARS-CoV-2" OR "Coronavirus Disease 2019 Virus" OR "Wuhan Seafood Market Pneumonia Virus" OR "SARS-CoV-2 Virus" OR "SARS CoV 2 Virus" OR "SARS-CoV-2 Viruses" OR "Virus, SARS-CoV-2" OR "2019-nCoV" OR "COVID-19 Virus" OR "COVID 19 Virus" OR "COVID-19 Viruses" OR "Virus, COVID-19" OR "Wuhan Coronavirus" OR "Coronavirus, Wuhan" OR "SARS Coronavirus 2" OR "Coronavirus 2, SARS" OR "Severe Acute Respiratory Syndrome Coronavirus 2" OR "2019 Novel Coronavirus" OR "2019 Novel Coronaviruses" OR "Coronavirus, 2019 Novel" OR "Novel Coronavirus, 2019" OR "2019 nCOV" OR "2019 severe acute respiratory syndrome coronavirus 2" OR "HCoV-19" OR "Human coronavirus 2019" OR "nCoV-2019" OR "SARS2 (virus)" OR "SARS-related coronavirus 2" OR "Sever acute respiratory syndrome coronavirus 2" OR "Severe acute respiratory coronavirus 2" OR "Severe acute respiratory syndorme coronavirus 2" OR "severe acute respiratory syndrome 2 virus" OR "severe acute respiratory syndrome corona virus 2" OR "severe acute respiratory syndrome coronavirus 2019" OR "Severe acute respiratory syndrome coronoavirus 2" OR "Severe acute respiratory syndrome coronvirus 2" OR "severe acute respiratory syndrome CoV-2 virus" OR "Severe acute respiratory syndrome related coronavirus 2" OR "Severe acute respiratory syndrome virus 2" OR "Severe acute respiratoy syndrome coronavirus 2" OR "2019 new coronavirus" OR "novel 2019 coronavirus" OR "novel coronavirus-19").mp.

#5	1 OR 2 OR 3 OR 4
#6	("pediatric multisystem inflammatory disease, COVID-19 related" OR "pediatric multisystem inflammatory syndrome, SARS-CoV-2 related" OR "pediatric multisystem inflammatory syndrome, COVID-19 related" OR "pediatric multi-system inflammatory syndrome, COVID-19 related" OR "pediatric multi-system inflammatory syndrome, SARS-CoV-2 related" OR "MISC associated with COVID-19" OR "multisystem inflammatory syndrome, pediatric, COVID-19 related" OR "multi-system inflammatory disease, pediatric, COVID-19 related" OR "multi-system inflammatory syndrome, pediatric, COVID-19 related" OR "PIMS-TS" OR "multisystem inflammatory syndrome in children MIS-C associated with COVID-19" OR "MIS-C associated with COVID-19" OR "pediatric multi-system inflammatory disease, COVID-19 related" OR "multisystem inflammatory disease, pediatric, COVID-19 related" OR "pediatric inflammatory multisystem syndrome" OR "MIS-C multisystem inflammatory syndrome in children" OR "multisystem inflammatory syndrome in children" OR "pediatric multisystem inflammatory syndrome" OR "childhood multisystem inflammatory syndrome" OR "COVID-19 associated Kawasaki-like multisystem inflammatory disease" OR "COVID-19 associated multisystem inflammatory syndrome" OR "COVID-19 Kawasaki-like syndrome" OR "COVID-19 related multisystem inflammatory syndrome" OR "Kawa-COVID-19" OR "Kawasaki like disease" OR "Kawasaki-like multisystem inflammatory syndrome" OR "MIS-C (multisystem inflammatory syndrome in children)" OR "multi-inflammatory syndrome in children" OR "multiorgan inflammatory syndrome associated with SARS-CoV-2" OR "multisystem inflammation syndrome in children" OR "multisystem inflammatory syndrome associated with SARS-CoV-2" OR "multi-system inflammatory syndrome in children" OR "paediatric inflammatory multisystem syndrome temporally associated with severe acute respiratory syndrome coronavirus 2" OR "paediatric multisystem inflammatory syndrome" OR "paediatric multi-system inflammatory syndrome" OR "paediatric multisystem inflammatory syndrome temporally associated with COVID-19" OR "paediatric multisystem inflammatory syndrome temporally associated with SARS-CoV-2" OR "pediatric inflammatory multisystem syndrome temporally associated with SARS-CoV-2" OR "pediatric inflammatory multisystem syndrome temporally associated with severe acute respiratory syndrome coronavirus 2" OR "pediatric multi-inflammatory syndrome" OR "pediatric multi-inflammatory syndrome mimicking Kawasaki disease" OR "pediatric multisystem inflammatory disease" OR "pediatric multi-system inflammatory syndrome" OR "pediatric multisystem inflammatory syndrome associated with COVID-19" OR "pediatric multisystem inflammatory syndrome temporally associated with COVID-19" OR "pediatric multisystem inflammatory syndrome temporally associated with SARS-CoV-2" OR "PMIS-TS" OR "SARS-CoV-2 associated multisystem inflammatory syndrome" OR "SARS-CoV-2 induced Kawasaki-like hyperinflammatory syndrome" OR "SARS-CoV-2 mimicking Kawasaki disease" OR "pediatric multi-system inflammatory disease" OR "MISC" OR "MIS-C" OR "multisystem inflammatory syndrome" OR "multi-system inflammatory syndrome" OR "multisystem inflammatory disease" OR "multi-system inflammatory disease").mp.
#7	5 AND 6
#8	exp interleukin 1 receptor blocking agent/
#9	("Interleukin 1 Receptor Antagonist Protein" OR "IL1 Febrile Inhibitor" OR "Febrile Inhibitor, IL1" OR "IL-1Ra" OR "Urine-Derived IL1 Inhibitor" OR "IL1 Inhibitor, Urine-Derived" OR "Urine Derived IL1 Inhibitor" OR "IL-1 Inhibitor, Urine" OR "IL 1 Inhibitor, Urine" OR "Urine IL-1 Inhibitor" OR "Interleukin 1 Inhibitor, Urine" OR "Antril" OR "Kineret" OR "Anakinra" OR "recombinant interleukin 1 receptor antagonist" OR "recombinant interleukin 1 receptor blocker" OR "recombinant interleukin 1 receptor blocking agent").mp.
#10	(interleukin-1 inhibitor* OR IL-1 inhibitor* OR "interleukin-1 inhibitor" OR "IL-1 inhibitor" OR "interleukin-1 receptor antagonist" OR "IL-1 receptor antagonist").mp.
#11	exp infliximab/

#12	("Infliximab" OR "Monoclonal Antibody cA2" OR "cA2, Monoclonal Antibody" OR "MAb cA2" OR "Infliximab-abda" OR "Renflexis" OR "Infliximab-dyyb" OR "Inflectra" OR "Remicade" OR "abp 710" OR "abp710" OR "avakine" OR "avsola" OR "flixabi" OR "gp 1111" OR "gp1111" OR "inflectra" OR "infliximab abda" OR "infliximab axxq" OR "infliximab dyyb" OR "infliximab qbtx" OR "infliximab-abda" OR "infliximab-axxq" OR "infliximab-dyyb" OR "infliximab-qbtx" OR "ixifi" OR "pf 06438179" OR "pf 6438179" OR "pf06438179" OR "pf6438179" OR "remsima" OR "renflexis" OR "revellex" OR "ta 650" OR "ta650" OR "zessly").mp.
#13	exp tocilizumab/
#14	("Tocilizumab" OR "RHPM-1" OR "RG-1569" OR "R-1569" OR "MSB11456" OR "MSB-11456" OR "atlizumab" OR "monoclonal antibody, MRA" OR "RO-4877533" OR "Actemra" OR "Roactemra" OR "actemra 200" OR "lusinex" OR "r 1569" OR "r1569").mp.
#15	(interleukin-6 inhibitor* OR IL-6 inhibitor* OR "interleukin-6 inhibitor" OR "IL-6 inhibitor" OR "interleukin-6 receptor antagonist" OR "IL-6 receptor antagonist").mp.
#16	exp convalescent plasma/
#17	("COVID-19 serotherapy" OR "COVID19 serum therapy" OR "coronavirus disease-19 serotherapy" OR "COVID19 serotherapy" OR "coronavirus disease 2019 serotherapy" OR "COVID-19 serum therapy" OR "COVID-19 convalescent serum treatment" OR "SARS-CoV-2 convalescent sera treatment" OR "SARS-CoV-2 convalescent serum treatment" OR "Covid-19 convalescent sera treatment" OR "convalescent serum treatment for Covid-19" OR "COVID-19 hyperimmune globulin therapy" OR "COVID19 hyperimmune globulin therapy" OR "hyperimmune globulin therapy for COVID-19" OR "COVID-19 convalescent plasma treatment" OR "convalescent plasma treatment for Covid-19" OR "SARS-CoV-2 convalescent plasma treatment" OR "convalescent plasma" OR "convalescence phase plasma" OR "convalescence plasma" OR "convalescent human plasma" OR "convalescent immune plasma" OR "convalescent patient plasma" OR "convalescent phase plasma" OR "plasma from convalescent" OR "plasma from convalescents" OR "PlasmAr").mp.
#18	8 OR 9 OR 10 OR 11 OR 12 OR 13 OR 14 OR 15 OR 16 OR 17
#19	7 AND 18
#20	limit 19 to dc=20200301-20211231

3) Cochrane library CENTRAL

no.	Search strategy
#1	[mh "COVID-19"]
#2	"COVID-19":ti,ab,kw OR "COVID 19":ti,ab,kw OR "COVID-19 Virus Disease":ti,ab,kw OR "COVID 19 Virus Disease":ti,ab,kw OR "COVID-19 Virus Diseases":ti,ab,kw OR "Disease, COVID-19 Virus":ti,ab,kw OR "Virus Disease, COVID-19":ti,ab,kw OR "COVID-19 Virus Infection":ti,ab,kw OR "COVID 19 Virus Infection":ti,ab,kw OR "COVID-19 Virus Infections":ti,ab,kw OR "Infection, COVID-19 Virus":ti,ab,kw OR "Virus Infection, COVID-19":ti,ab,kw OR "2019-nCoV Infection":ti,ab,kw OR "2019 nCoV Infection":ti,ab,kw OR "2019-nCoV Infections":ti,ab,kw OR "Infection, 2019-nCoV":ti,ab,kw OR "Coronavirus Disease-19":ti,ab,kw OR "Coronavirus Disease 19":ti,ab,kw OR "2019-nCoV Disease":ti,ab,kw OR "2019 nCoV Disease":ti,ab,kw OR "2019-nCoV Diseases":ti,ab,kw OR "Disease, 2019-nCoV":ti,ab,kw OR "COVID19":ti,ab,kw OR "Coronavirus Disease 2019":ti,ab,kw OR "Disease 2019, Coronavirus":ti,ab,kw OR "SARS Coronavirus 2 Infection":ti,ab,kw OR "SARS-CoV-2 Infection":ti,ab,kw OR "Infection, SARS-CoV-2":ti,ab,kw OR "SARS CoV 2 Infection":ti,ab,kw OR "SARS-CoV-2 Infections":ti,ab,kw OR "COVID-19 Pandemic":ti,ab,kw OR "COVID 19 Pandemic":ti,ab,kw OR "COVID-19 Pandemics":ti,ab,kw OR "Pandemic, COVID-19":ti,ab,kw OR "2019 Novel Coronavirus Disease":ti,ab,kw OR "2019 Novel Coronavirus Infection":ti,ab,kw OR "coronavirus disease 2":ti,ab,kw OR "coronavirus disease 2019 pneumonia":ti,ab,kw OR "coronavirus infection 2019":ti,ab,kw OR "COVID":ti,ab,kw OR "COVID 19 induced pneumonia":ti,ab,kw OR "COVID 2019":ti,ab,kw OR "COVID-19 induced pneumonia":ti,ab,kw OR "COVID-19 pneumonia":ti,ab,kw OR "nCoV 2019 disease":ti,ab,kw OR "nCoV 2019 infection":ti,ab,kw OR "paucisymptomatic coronavirus disease 2019":ti,ab,kw OR "SARS coronavirus 2 pneumonia":ti,ab,kw OR "SARSCoV2 disease":ti,ab,kw OR "SARS-CoV2 disease":ti,ab,kw OR "SARS-CoV-2 disease":ti,ab,kw OR "SARSCoV2 infection":ti,ab,kw OR "SARS-CoV2 infection":ti,ab,kw OR "SARS-CoV-2 pneumonia":ti,ab,kw OR "severe acute respiratory syndrome 2":ti,ab,kw OR "severe acute respiratory syndrome 2 pneumonia":ti,ab,kw OR "severe acute respiratory syndrome coronavirus 2 infection":ti,ab,kw OR "severe acute respiratory syndrome coronavirus 2019 infection":ti,ab,kw OR "severe acute respiratory syndrome CoV-2 infection":ti,ab,kw OR "Wuhan coronavirus disease":ti,ab,kw OR "Wuhan coronavirus infection":ti,ab,kw OR "2019 novel coronavirus epidemic":ti,ab,kw OR "new coronavirus pneumonia":ti,ab,kw OR "novel coronavirus 2019 disease":ti,ab,kw OR "novel coronavirus 2019 infection":ti,ab,kw OR "novel coronavirus disease 2019":ti,ab,kw OR "novel coronavirus infected pneumonia":ti,ab,kw OR "novel coronavirus infection 2019":ti,ab,kw OR "novel coronavirus pneumonia":ti,ab,kw OR "2019nCoV":ti,ab,kw OR "19nCoV":ti,ab,kw OR "COVID19*":ti,ab,kw OR "SARSCOV-2":ti,ab,kw OR "SARSCOV2":ti,ab,kw OR "corona virus 2":ti,ab,kw OR "Wuhan":ti,ab,kw OR "Hubei":ti,ab,kw OR "new coronavirus":ti,ab,kw OR "novel coronavirus":ti,ab,kw OR "novel corona virus":ti,ab,kw OR "novel CoV":ti,ab,kw
#3	[mh "SARS-CoV-2"]
#4	"SARS-CoV-2":ti,ab,kw OR "Coronavirus Disease 2019 Virus":ti,ab,kw OR "Wuhan Seafood Market Pneumonia Virus":ti,ab,kw OR "SARS-CoV-2 Virus":ti,ab,kw OR "SARS CoV 2 Virus":ti,ab,kw OR "SARS-CoV-2 Viruses":ti,ab,kw OR "Virus, SARS-CoV-2":ti,ab,kw OR "2019-nCoV":ti,ab,kw OR "COVID-19 Virus":ti,ab,kw OR "COVID 19 Virus":ti,ab,kw OR "COVID-19 Viruses":ti,ab,kw OR "Virus, COVID-19":ti,ab,kw OR "Wuhan Coronavirus":ti,ab,kw OR "Coronavirus, Wuhan":ti,ab,kw OR "SARS Coronavirus 2":ti,ab,kw OR "Coronavirus 2, SARS":ti,ab,kw OR "Severe Acute Respiratory Syndrome Coronavirus 2":ti,ab,kw OR "2019 Novel Coronavirus":ti,ab,kw OR "2019 Novel Coronaviruses":ti,ab,kw OR "Coronavirus, 2019 Novel":ti,ab,kw OR "Novel Coronavirus, 2019":ti,ab,kw OR "2019 nCOV":ti,ab,kw OR "2019 severe acute respiratory syndrome coronavirus 2":ti,ab,kw OR "HCoV-19":ti,ab,kw OR "Human coronavirus 2019":ti,ab,kw OR "nCoV-2019":ti,ab,kw OR "SARS2 (virus)":ti,ab,kw OR "SARS-related coronavirus 2":ti,ab,kw OR "Sever acute respiratory syndrome coronavirus 2":ti,ab,kw OR "Severe acute respiratory coronavirus 2":ti,ab,kw OR "Severe acute respiratory syndorme coronavirus 2":ti,ab,kw OR "severe acute respiratory syndrome 2 virus":ti,ab,kw OR "severe acute respiratory syndrome corona virus 2":ti,ab,kw OR "severe acute respiratory syndrome coronavirus 2019":ti,ab,kw OR "Severe acute respiratory syndrome coronoavirus 2":ti,ab,kw OR "Severe acute respiratory syndrome coronvirus 2":ti,ab,kw OR "severe acute respiratory syndrome CoV-2 virus":ti,ab,kw OR "Severe acute respiratory syndrome related coronavirus 2":ti,ab,kw OR "Severe acute respiratory syndrome virus 2":ti,ab,kw OR "Severe acute respiratoy syndrome coronavirus 2":ti,ab,kw OR "2019 new coronavirus":ti,ab,kw OR "novel 2019 coronavirus":ti,ab,kw OR "novel coronavirus-19":ti,ab,kw

#5	#1 OR #2 OR #3 OR #4
#6	"pediatric multisystem inflammatory disease, COVID-19 related":ti,ab,kw OR "pediatric multisystem inflammatory syndrome, SARS-CoV-2 related":ti,ab,kw OR "pediatric multisystem inflammatory syndrome, COVID-19 related":ti,ab,kw OR "pediatric multi-system inflammatory syndrome, COVID-19 related":ti,ab,kw OR "pediatric multi-system inflammatory syndrome, SARS-CoV-2 related":ti,ab,kw OR "MISC associated with COVID-19":ti,ab,kw OR "multisystem inflammatory syndrome, pediatric, COVID-19 related":ti,ab,kw OR "multi-system inflammatory disease, pediatric, COVID-19 related":ti,ab,kw OR "multi-system inflammatory syndrome, pediatric, COVID-19 related":ti,ab,kw OR "PIMS-TS":ti,ab,kw OR "multisystem inflammatory syndrome in children MIS-C associated with COVID-19":ti,ab,kw OR "MIS-C associated with COVID-19":ti,ab,kw OR "pediatric multi-system inflammatory disease, COVID-19 related":ti,ab,kw OR "multisystem inflammatory disease, pediatric, COVID-19 related":ti,ab,kw OR "pediatric inflammatory multisystem syndrome":ti,ab,kw OR "MIS-C multisystem inflammatory syndrome in children":ti,ab,kw OR "multisystem inflammatory syndrome in children":ti,ab,kw OR "pediatric multisystem inflammatory syndrome":ti,ab,kw OR "childhood multisystem inflammatory syndrome":ti,ab,kw OR "COVID-19 associated Kawasaki-like multisystem inflammatory disease":ti,ab,kw OR "COVID-19 associated multisystem inflammatory syndrome":ti,ab,kw OR "COVID-19 Kawasaki-like syndrome":ti,ab,kw OR "COVID-19 related multisystem inflammatory syndrome":ti,ab,kw OR "Kawa-COVID-19":ti,ab,kw OR "Kawasaki like disease":ti,ab,kw OR "Kawasaki-like multisystem inflammatory syndrome":ti,ab,kw OR "MIS-C (multisystem inflammatory syndrome in children)":ti,ab,kw OR "multi-inflammatory syndrome in children":ti,ab,kw OR "multiorgan inflammatory syndrome associated with SARS-CoV-2":ti,ab,kw OR "multisystem inflammation syndrome in children":ti,ab,kw OR "multisystem inflammatory syndrome associated with SARS-CoV-2":ti,ab,kw OR "multi-system inflammatory syndrome in children":ti,ab,kw OR "paediatric inflammatory multisystem syndrome temporally associated with severe acute respiratory syndrome coronavirus 2":ti,ab,kw OR "paediatric multisystem inflammatory syndrome":ti,ab,kw OR "paediatric multi-system inflammatory syndrome":ti,ab,kw OR "paediatric multisystem inflammatory syndrome temporally associated with COVID-19":ti,ab,kw OR "paediatric multisystem inflammatory syndrome temporally associated with SARS-CoV-2":ti,ab,kw OR "pediatric inflammatory multisystem syndrome temporally associated with SARS-CoV-2":ti,ab,kw OR "pediatric inflammatory multisystem syndrome temporally associated with severe acute respiratory syndrome coronavirus 2":ti,ab,kw OR "pediatric multi-inflammatory syndrome":ti,ab,kw OR "pediatric multi-inflammatory syndrome mimicking Kawasaki disease":ti,ab,kw OR "pediatric multisystem inflammatory disease":ti,ab,kw OR "pediatric multi-system inflammatory syndrome":ti,ab,kw OR "pediatric multisystem inflammatory syndrome associated with COVID-19":ti,ab,kw OR "pediatric multisystem inflammatory syndrome temporally associated with COVID-19":ti,ab,kw OR "pediatric multisystem inflammatory syndrome temporally associated with SARS-CoV-2":ti,ab,kw OR "PMIS-TS":ti,ab,kw OR "SARS-CoV-2 associated multisystem inflammatory syndrome":ti,ab,kw OR "SARS-CoV-2 induced Kawasaki-like hyperinflammatory syndrome":ti,ab,kw OR "SARS-CoV-2 mimicking Kawasaki disease":ti,ab,kw OR "pediatric multi-system inflammatory disease":ti,ab,kw OR "MISC":ti,ab,kw OR "MIS-C":ti,ab,kw OR "multisystem inflammatory syndrome":ti,ab,kw OR "multi-system inflammatory syndrome":ti,ab,kw OR "multisystem inflammatory disease":ti,ab,kw OR "multi-system inflammatory disease":ti,ab,kw
#7	#5 AND #6
#8	[mh "Interleukin 1 Receptor Antagonist Protein"]
#9	"Interleukin 1 Receptor Antagonist Protein":ti,ab,kw OR "IL1 Febrile Inhibitor":ti,ab,kw OR "Febrile Inhibitor, IL1":ti,ab,kw OR "IL-1Ra":ti,ab,kw OR "Urine-Derived IL1 Inhibitor":ti,ab,kw OR "IL1 Inhibitor, Urine-Derived":ti,ab,kw OR "Urine Derived IL1 Inhibitor":ti,ab,kw OR "IL-1 Inhibitor, Urine":ti,ab,kw OR "IL 1 Inhibitor, Urine":ti,ab,kw OR "Urine IL-1 Inhibitor":ti,ab,kw OR "Interleukin 1 Inhibitor, Urine":ti,ab,kw OR "Antril":ti,ab,kw OR "Kineret":ti,ab,kw OR "Anakinra":ti,ab,kw OR "recombinant interleukin 1 receptor antagonist":ti,ab,kw OR "recombinant interleukin 1 receptor blocker":ti,ab,kw OR "recombinant interleukin 1 receptor blocking agent":ti,ab,kw

#10	"interleukin-1 inhibitor*":ti,ab,kw OR "IL-1 inhibitor*":ti,ab,kw OR "interleukin-1 inhibitor":ti,ab,kw OR "IL-1 inhibitor":ti,ab,kw OR "interleukin-1 receptor antagonist":ti,ab,kw OR "IL-1 receptor antagonist":ti,ab,kw
#11	[mh "Infliximab"]
#12	"Infliximab":ti,ab,kw OR "Monoclonal Antibody cA2":ti,ab,kw OR "cA2, Monoclonal Antibody":ti,ab,kw OR "MAb cA2":ti,ab,kw OR "Infliximab-abda":ti,ab,kw OR "Renflexis":ti,ab,kw OR "Infliximab-dyyb":ti,ab,kw OR "Inflectra":ti,ab,kw OR "Remicade":ti,ab,kw OR "abp 710":ti,ab,kw OR "abp710":ti,ab,kw OR "avakine":ti,ab,kw OR "avsola":ti,ab,kw OR "flixabi":ti,ab,kw OR "gp 1111":ti,ab,kw OR "gp1111":ti,ab,kw OR "inflectra":ti,ab,kw OR "infliximab abda":ti,ab,kw OR "infliximab axxq":ti,ab,kw OR "infliximab dyyb":ti,ab,kw OR "infliximab qbtx":ti,ab,kw OR "infliximab-abda":ti,ab,kw OR "infliximab-axxq":ti,ab,kw OR "infliximab-dyyb":ti,ab,kw OR "infliximab-qbtx":ti,ab,kw OR "ixifi":ti,ab,kw OR "pf 06438179":ti,ab,kw OR "pf 6438179":ti,ab,kw OR "pf06438179":ti,ab,kw OR "pf6438179":ti,ab,kw OR "remsima":ti,ab,kw OR "renflexis":ti,ab,kw OR "revellex":ti,ab,kw OR "ta 650":ti,ab,kw OR "ta650":ti,ab,kw OR "zessly":ti,ab,kw
#13	"Tocilizumab":ti,ab,kw OR "RHPM-1":ti,ab,kw OR "RG-1569":ti,ab,kw OR "R-1569":ti,ab,kw OR "MSB11456":ti,ab,kw OR "MSB-11456":ti,ab,kw OR "atlizumab":ti,ab,kw OR "monoclonal antibody, MRA":ti,ab,kw OR "RO-4877533":ti,ab,kw OR "Actemra":ti,ab,kw OR "Roactemra":ti,ab,kw OR "actemra 200":ti,ab,kw OR "lusinex":ti,ab,kw OR "r 1569":ti,ab,kw OR "r1569":ti,ab,kw
#14	"interleukin-6 inhibitor*":ti,ab,kw OR "IL-6 inhibitor*":ti,ab,kw OR "interleukin-6 inhibitor":ti,ab,kw OR "IL-6 inhibitor":ti,ab,kw OR "interleukin-6 receptor antagonist":ti,ab,kw OR "IL-6 receptor antagonist":ti,ab,kw
#15	"COVID-19 serotherapy":ti,ab,kw OR "COVID19 serum therapy":ti,ab,kw OR "coronavirus disease-19 serotherapy":ti,ab,kw OR "COVID19 serotherapy":ti,ab,kw OR "coronavirus disease 2019 serotherapy":ti,ab,kw OR "COVID-19 serum therapy":ti,ab,kw OR "COVID-19 convalescent serum treatment":ti,ab,kw OR "SARS-CoV-2 convalescent sera treatment":ti,ab,kw OR "SARS-CoV-2 convalescent serum treatment":ti,ab,kw OR "Covid-19 convalescent sera treatment":ti,ab,kw OR "convalescent serum treatment for Covid-19":ti,ab,kw OR "COVID-19 hyperimmune globulin therapy":ti,ab,kw OR "COVID19 hyperimmune globulin therapy":ti,ab,kw OR "hyperimmune globulin therapy for COVID-19":ti,ab,kw OR "COVID-19 convalescent plasma treatment":ti,ab,kw OR "convalescent plasma treatment for Covid-19":ti,ab,kw OR "SARS-CoV-2 convalescent plasma treatment":ti,ab,kw OR "convalescent plasma":ti,ab,kw OR "convalescence phase plasma":ti,ab,kw OR "convalescence plasma":ti,ab,kw OR "convalescent human plasma":ti,ab,kw OR "convalescent immune plasma":ti,ab,kw OR "convalescent patient plasma":ti,ab,kw OR "convalescent phase plasma":ti,ab,kw OR "plasma from convalescent":ti,ab,kw OR "plasma from convalescents":ti,ab,kw OR "PlasmAr":ti,ab,kw
#16	#8 OR #9 OR #10 OR #11 OR #12 OR #13 OR #14 OR #15
#17	#5 AND #6 AND #16
#18	#17 with Cochrane Library publication date from Mar 2020 to Dec 2021

4) KMBASE

no.	Search strategy
#1	([ALL=COVID-19] OR [ALL=COVID19] OR [ALL=coronavirus] OR [ALL=SARS-CoV-2] OR [ALL=Severe acute respiratory syndrome coronavirus 2]) AND ([ALL=Multisystem Inflammatory Syndrome] OR [ALL=multisystem inflammatory disease] OR [ALL=MIS-C])
#2	([ALL=COVID-19] OR [ALL=COVID19] OR [ALL=coronavirus] OR [ALL=SARS-CoV-2] OR [ALL=Severe acute respiratory syndrome coronavirus 2]) AND ([ALL=소아 다기관 염증 증후군 [a]] OR [ALL=소아 다기관 염증증후군 [a]])
#3	([ALL=코로나-19[b]] OR [ALL=코로나 19 [b]]) OR [ALL=코로나][c]) AND ([ALL=Multisystem Inflammatory Syndrome] OR [ALL=multisystem inflammatory disease] OR [ALL=MIS-C])
#4	([ALL=코로나-19 [b]] OR [ALL=코로나 19 [b]]) OR [ALL=코로나] [c]) AND ([ALL=소아 다기관 염증 증후군 [a]] OR [ALL=소아 다기관 염증증후군 [a]])
#5	#1 OR #2 OR #3 OR #4

[a] MIS-C in Korean

[b] COVID-19 in Korean

[c] Coronavirus in Korean

Figure S1. Risk of bias graph

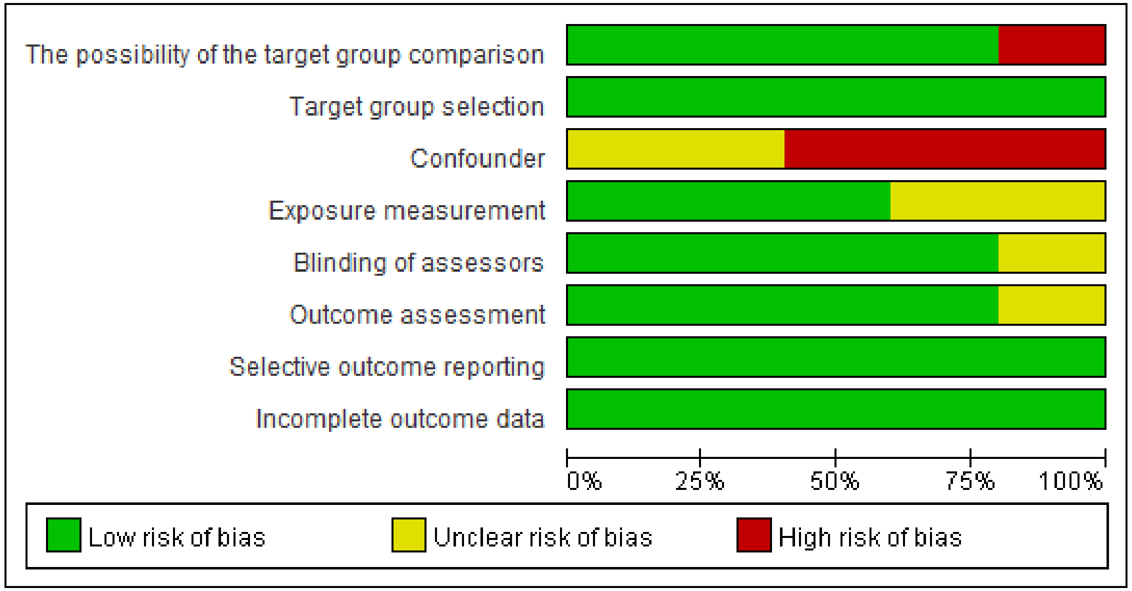

Table S3. Risk of bias summary

	The possibility of the target group comparison	Target group selection	Confounder	Exposure measurement	Blinding of assessors	Outcome assessment	Selective outcome reporting	Incomplete outcome data
Brisca 2021	−	+	−	?	+	?	+	+
Campanello 2022	+	+	−	+	+	+	+	+
Çelikel 2021	+	+	?	+	+	+	+	+
Cole 2021	+	+	?	+	?	+	+	+
Sozeri 2021	+	+	−	?	+	+	+	+

Table S4. Summary of sequence of immunomodulatory treatment

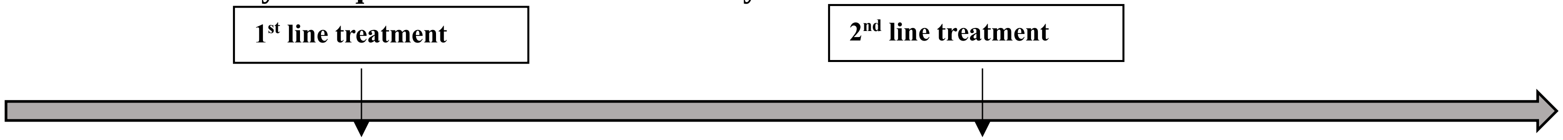

<table>
<tr><th>Source</th><th>First-line treatment (N,%)</th><th>Indication for second-line treatment (N,%)</th><th>Comment</th></tr>
<tr><td>Celikel et al. [8], 2021</td><td>IVIg and corticosteroid (10,30%)</td><td>Biologics given if refractory within 24 hours (23, 70%)</td><td></td></tr>
<tr><td>Cole et al. [9], 2021</td><td>IVIg or IVIg and infliximab</td><td>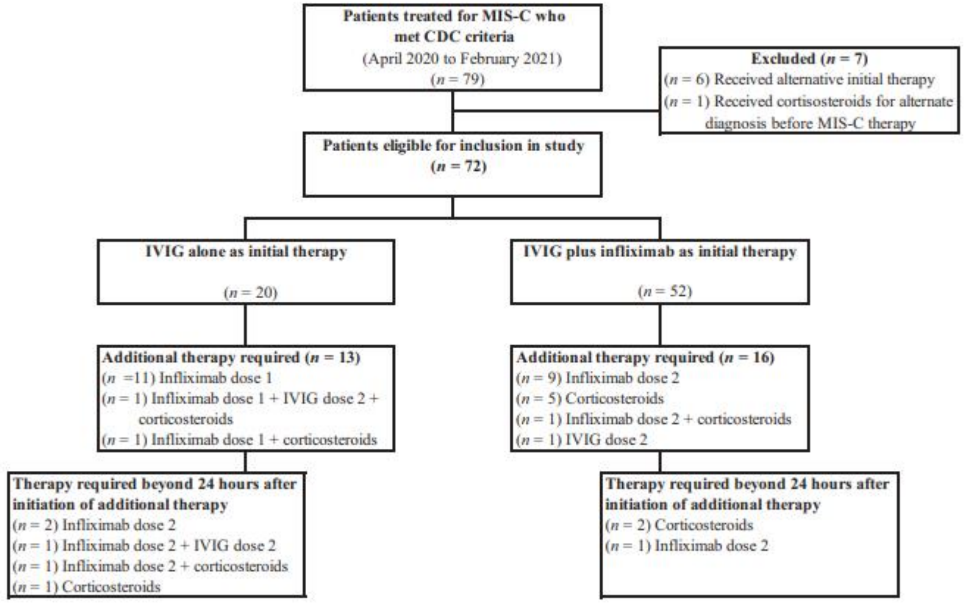

FIGURE 1
Study schematic for patients diagnosed with MIS-C by CDC guidelines who received treatment. Alternative initial therapies included IVIG plus corticosteroids, infliximab, and/or anakinra.</td><td>Figure 1 from the Cole et al. study was attached for better illustration of the treatment process.</td></tr>
</table>

Gruber et al. [2], 2020	IVIg or biologic immunomodulator	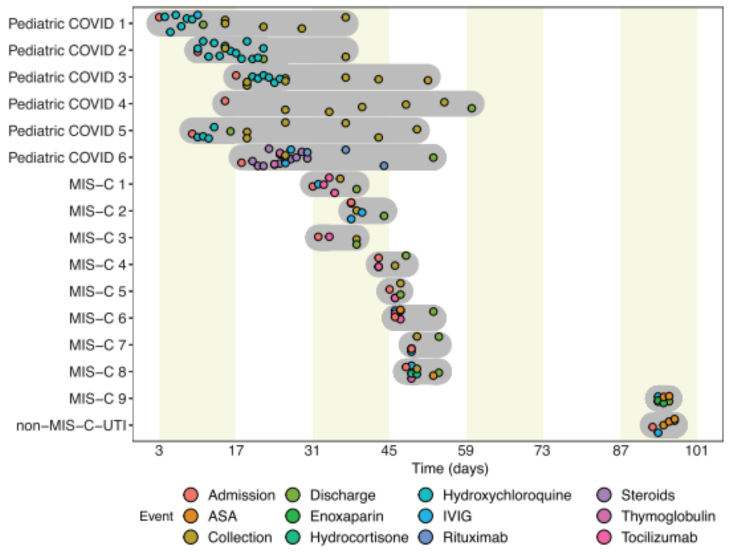<br><br>**Figure S1. Chest X-Ray Images from Three MIS-C Patients and Patient Clinical Timelines, Related to Table 1**<br>(A) MIS-C 1: Reactive airway disease with no evidence of pneumonia or atelectasis. (B) MIS-C 3: Cardiomegaly, retrocardiac opacity, and bilateral pleural effusion. (C) MIS-C 5: Mild bilateral, right greater than left, patchy and hazy pulmonary opacities in a basilar distribution. (D) Disease and treatment timeline for the MIS-C patients (N=9), pediatric COVID patients (N=6) and non-MIS-C UTI patient (N=1). Shaded regions represent complete sampling period (inclusive for hospital admission and discharge) for respective patients. Plot shading (beige) correspond to biweekly intervals.	There was no standardized timeline or order of the treatment. 2 patients received IVIG as first-line, while the remaining were administered with biological immunomodulator first.<br>Figure S1 from Gruber *et al*. study was attached for better illustration of the time line.
Papadopoulou et al. [10], 2021	IVIg and corticosteroid	If refractory (specific indication and time limit not specified) to 1st line treatment	
Abdel-Haq et al. [11], 2021	IVIg	2nd IVIg was administered if fever did not subside in 48 hours (2→ if refractory, infliximab was used (12, 92%)	
Lee et al. [12], 2020	IVIg (20,71%) And/or corticosteroid (1,4%)	If refractory to initial treatment, anakinra was administered (5, 18%).	
Campanello et al. [13], 2022	IVIg and corticosteroid (25,100%)	Anakinra was administered in severe cases and/or refractory to initial treatment (8, 32%)	

Brisca et al. [14], 2021	IVIg or<br>IVIg and corticosteroid and anakinra (6) (if symptoms were severe, cardiac dysfunction, lab indicative of macrophage activation syndrome)	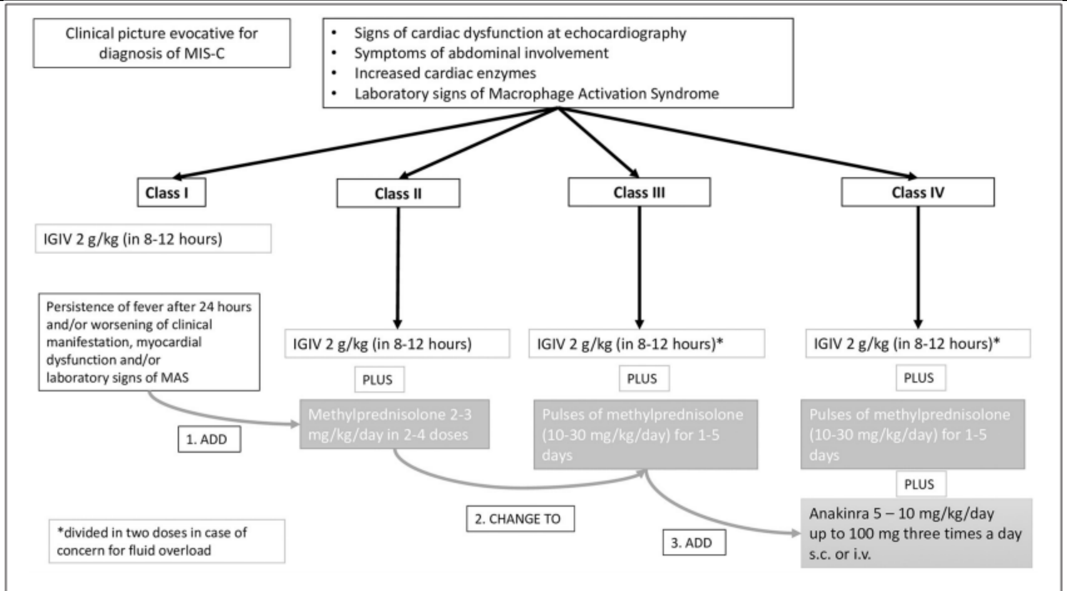<br>**FIGURE 1** | Multistep antinflammatory treatment protocol for MIS-C.	Figure 1 from the Brisca *et al*. study was attached for better illustration of the treatment process.
Sozeri et al. [15], 2021	IVIg (58,85%)<br>IVIg and corticosteroid (20,30%)<br>Anakinra (17, 25%)<br>Tocilizumab (1, 2%)	No specific indication or time line delineated for corticosteroid or biologic immunomodulatory use	
Savas Sen et al.[16], 2020	IVIG	If fever persisted and/or inflammatory markers were rising despite IVIG, corticosteroid was used. If severe or refractory shock, high dose methylprednisolone and anakinra combination therapy was used (9, 20%)	As result, IVIg only was used in 11 patients (24%), IVIg and corticosteroid in 12 patients (27%), and IVIg, corticosteroid, and anakinra combination in 6 patients (13%).

Table S5. Comparison of Cole *et al*. and Celikel *et al*. studies

Parameters	Celikel et al. [8], 2021 (N = 33)	Cole et al. [9], 2021 (N = 72)
Study group	IVIg + Corticosteroid + Biologic(anakinra or tocilizumab) (23,70%)	IVIg +Infliximab (52, 28%)
Control group	IVIG + Corticosteroid (10,30%)	IVIG alone (20, 72%)
ICU admission (n,%)	All severe MIS-C cases admitted to ICU were included (33, 100%).	IVIg alone (8,40%) vs. IVIg+infliximab (34,65%)
Initial treatment	IVIG and corticosteroid (10,30%)	IVIG or IVIG and infliximab
Second-line or additional treatment indication	Anakinra was given if refractory to initial treatment within 24 hours (23, 70%) and if refractory to anakinra treatment for 7 days, tocilizumab was used (2, 6%)	Administration of immunomodulatory agent (infliximab, corticosteroids, 2nd dose of IVIG, or combination) >24 hours after treatment initiation if fever persisted more than 24 hours after completion of initial therapy, continued need for vasoactive medication, worsening echocardiographic findings, and/or laboratory evidence of persistently high inflammation
**Treatment outcomes**		
Change in C-reactive protein (CRP, mg/L,IQR) after treatment	Study group: 211 (83-357) → after 1st week of treatment 4.4 (0.5-146) ($P < 0.001$)<br>Control group: not shown	Study group: 70% (-79 to -49) decrease after ≥48 to <72 hours after treatment<br>Control: -5 (-41 to 57) decrease
LVEF (median,%)	Study group: 55 (30-65) → after 1st week of treatment 69 (44-78) (P<0.001)	The number of cases of new or worsened LV dysfunction (newly developed EF<55% or decrease in EF by ≥5% at 24 hours after treatment)<br>Study group: 2(4%) vs. Control: 4 (20%) $P = 0.05$
Hospital length of stay (days, median)	15 (6-21)	Study group: 3.7 (3.2-4.5) vs. Control : 4.5 (3.4-5.9)
ICU length of stay (days, median)	7 (2-15)	Study group: 1.5 (1.1-2.1) vs. Control: 3.3(2.2-3.8)
